# Incidence, risk factors and prevention of hypothyroidism following laryngectomy: a systematic review and meta-analysis

**DOI:** 10.1308/rcsann.2025.0001

**Published:** 2025-07-15

**Authors:** JY Tan, E Westwood, O Edafe

**Affiliations:** ^1^ NHS Greater Glasgow and Clyde, UK; ^2^ Yorkshire and Humber School of Public Health, UK; ^3^ University of Sheffield, UK

**Keywords:** (MeSH Unique ID): Hypothyroidism (D007037), Laryngectomy (D007825), Incidence (D015994), Risk factor (D012307), Primary prevention (D011322)

## Abstract

**Introduction:**

Hypothyroidism following laryngectomy is a well-recognised complication. The symptoms are multisystemic and can cause significant morbidity in patients. We aim to characterise the incidence of hypothyroidism following laryngectomy, and identify risk factors and preventative measures.

**Methods:**

A systematic search of EMBASE and PubMed was performed. We appraised relevant articles as per the predefined eligibility criteria. A quality assessment of the included studies was done. A meta-analysis was performed to evaluate the association between reported risk factors and hypothyroidism.

**Results:**

Forty articles were included. This encompassed a total of 3,061 patients with a median age of 61 years. Overall incidence of hypothyroidism was 50% (interquartile range: 38.3–75.7). The following factors were significantly associated with hypothyroidism: hemithyroidectomy, odds ratio (OR) 4.84 (95% confidence interval [CI] 3.46–6.77); radiotherapy, OR 4.4 (95% CI 2.29–8.43); and neck dissection, OR 2.63 (95% CI 1.56–4.44). Age, sex, chemotherapy and tumour stage were not significant in the meta-analysis. Preventative measures were based on reducing the extent of thyroid dissection, attention to the preservation of blood supply, and pre- and postoperative thyroid function test monitoring.

**Conclusions:**

A significant proportion of patients develop hypothyroidism following laryngectomy. Utilising known risk factors may direct a preventative measure. Further well-designed multicentre observational studies exploring preventative measures including reducing hemithyroidectomy, monitoring intervals of thyroid function and utility of routine thyroxine replacement are required.

## Introduction

Hypothyroidism is a well-recognised complication of laryngectomy; however, reported incidence varies.^1–5^ The extent of thyroid dissection and the requirement for multimodal treatment regimens including radiotherapy differ between patients, and have long been linked with this variance. Several demographic factors such an age and sex are also considered influential.^6,7^ Hypothyroidism itself can be broadly divided into overt hypothyroidism and subclinical hypothyroidism. Both are defined, investigated and managed in several ways, adding further to heterogeneity in the literature.

Symptoms of hypothyroidism are insidious and multisystemic, resulting in significant morbidity for the patient. Classic features include fatigue, cold intolerance, weight gain, constipation and dry skin, but the signs and symptoms are non-specific.^8^ In the postoperative context, hypothyroidism is associated with poor wound healing, complications such as salivary fistula formation, cardiac morbidity and depression.^9^ Mechanisms of developing postoperative hypothyroidism include concomitant thyroidectomy, iatrogenic injuries or sacrificed blood vessels of the thyroid gland during surgery. Both preoperative and postoperative neck radiation may also play a role in the development of hypothyroidism, likely confounded by advanced disease and the extent of surgical treatment.^6,10^

We aim to characterise the incidence of hypothyroidism following laryngectomy, and identify risk factors and preventative measures.

## Methods

We included all observational or interventional studies that evaluated the incidence, risk or prevention of hypothyroidism following laryngectomy. We excluded articles with fewer than ten patients, case reports, reviews, commentaries and articles not available in the English language.

A systematic literature search of EMBASE and PubMed was undertaken to publication date (8 August 2023). We restricted the search to articles available in the English language. We used the following search strategy: (thyroid replacement OR underactive thyroid OR hypothyroid OR hypothyroidism OR thyroid hormone OR thyroxine OR levothyroxine) AND (laryngectomy OR pharyngolaryngectomy). A further search update was done post analysis on 18 December 2024, and one article met our criteria as stated; the findings are presented in the discussion.

Two authors (EW and OE) screened titles and abstracts according to the eligibility criteria. Any query over the selection process was discussed between the two authors (EW and OE).

One author (EW) independently evaluated texts using a standardised data collection proforma. All extracted data were checked for accuracy by another author (JYT). Any discrepancies were discussed and amended accordingly.

The following data were collected in individual studies: study characteristics and population; extent of surgery; radiotherapy; definition of hypothyroidism; rates, risk factors and prevention of hypothyroidism. Participant demographics and underlying pathology, the extent of dissections performed and the use of any additional treatment modalities were recorded.

Murad’s tool was used to assess the quality of the included single-arm observational studies (Appendix 1 – available online).^11^ Cochrane collaboration’s tool was used to assess risk of bias in randomised controlled trials (Appendix 2 – available online).^12^

### Statistical analysis

Meta-analysis was done to evaluate the association between reported risk factors and hypothyroidism where complete data were available. A random effect model was used because of the variability in definitions, disease and treatment extent across studies. The *I*^2^ statistic was used to evaluate the degree of heterogeneity in the meta-analysis. RStudio with meta package was used to perform the meta-analysis. Forest plots for the individual risk factors are included in this review. Summary statistics of categorical variables were summarised as count and percentages. The median age (interquartile range [IQR]) of the review population was calculated. Where possible, rates of hypothyroidism were calculated and summarised across studies.

Because the review was based on previously published studies, no ethical approval or patient consent were required.

## Results

**Figure 1:**
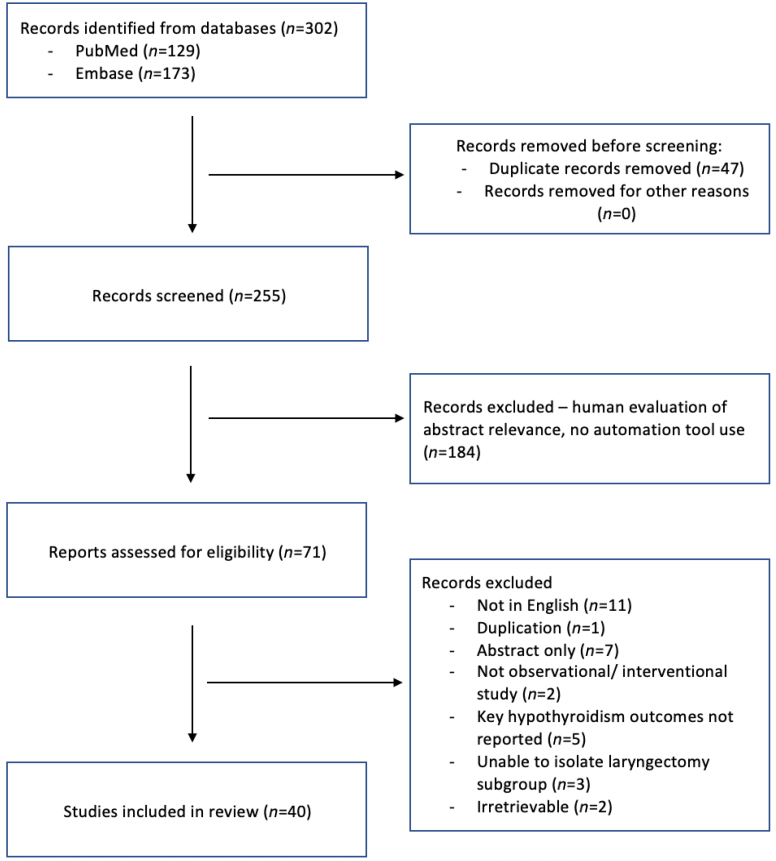
PRISMA flow chart outlining search strategy used for this systematic review

Forty studies were included in this review. Figure 1 shows the PRISMA flow chart outlining the search strategy used for this systematic review. The study designs were as follows: retrospective, 26; prospective, 13; randomised controlled trial, 1. The median (IQR) quality score of observational studies included was 3 (2–3). The one randomised controlled trial showed a high risk of bias on assessment with the Cochrane risk of bias tool for randomised controlled trials. The systematic review’s objectives were studied as the primary outcome in 29 studies and as the secondary outcome in 11 studies.

The systematic review included a total of 3,061 patients who had total laryngectomy. In total, 20 of 40 studies established thyroid status preoperatively. A definition of hypothyroidism using thyroid-stimulating hormone (TSH) or free T4 (fT4) measurements was reported in 29 studies; 11 studies did not record TSH or free T4 measurements.

Table 1 shows the demographics of patients in the review population and treatment details.

**Table 1:** Demographics of review population and treatment details

Median age, years	61
Male to female ratio	87:13
Patients on adjuvant or neoadjuvant radiotherapy, *n* (%)	1,766 (63)
Primary treatment	1,252 patients
Salvage treatment	813 patients
Extent of thyroid dissections, *n* (%)	
Hemithyroidectomy	1,418 (52)
Total thyroidectomy	425 (15)
No thyroid dissection	897 (33)

### Incidence

The median (IQR) reported incidence of hypothyroidism was 50% (38.3–75.7). The median incidence of subclinical and clinical hypothyroidism across all studies was 27% (range 10–54%) and 24% (range 3.3–64.7%), respectively.

### Risk factors

Nineteen studies evaluated the risk factors of hypothyroidism following laryngectomy. The risk factors evaluated were age and sex, hemithyroidectomy or total thyroidectomy, tumour stage, radiotherapy, chemotherapy and neck dissection. Table 2 shows risk factors evaluated for their association with postoperative hypothyroidism. A meta-analysis was carried out to evaluate the association between reported risk factors and hypothyroidism following laryngectomy.

**Table 2:** Risk factors evaluated for their association with postoperative hypothyroidism

Risk factors	Reduced risk	No risk	Increased risk
Older patients	Plaat *et al*^7^ (*p* < 0.001)	Alkan *et al*,^14^ Garcia-Serra *et al*,^18^ Ho *et al*,^4^ Qian *et al*,^20^ Lo Galbo *et al*,^5^ Ozawa *et al*,^22^ Smolarz *et al*,^35^ Tami *et al*,^23^ Viljoen *et al*^16^	Lo Galbo *et al*^21^
Female		Ho *et al*,^4^ Qian *et al*, ^20^ Lo Galbo *et* al,^5,10,21^ Ozawa *et al*,^22^ Plaat *et al*,^7^ Tami *et al*^23^	Gal *et al*,^6^ Léon *et al*^13^
Advanced tumours		Lo Galbo *et al*,^5,10,21^ Alkan *et al*,^14^ Léon *et al*,^13^ Viljoen *et al*,^16^ Tami *et al*,^23^ Qian *et al*,^20^ Ozawa *et al*^22^	Ho *et al*,^4^ Garcia-Serra *et al*,^18^ Rosko *et al*,^19^ Gal *et al*^6^
Hemithyroidectomy		Lo Galbo *et al* (hemithyroidectomy vs none),^21^ Tami *et al* (hemithyroidectomy vs none)^23^	Ho *et al* (hemithyroidectomy),^4^ Lo Galbo *et al*,^10^ Gal *et al* (total thyroidectomy),^6^ Plaat *et al* (hemithyroidectomy)^7^, Alkan *et al* (lobectomy),^14^ El-Sebai Ali *et al* (hemithyroidectomy),^15^ Léon *et al* (hemithyroidectomy),^13^ Viljoen *et al* (hemithyroidectomy),^16^ Turgut *et al* (hemithyroidectomy)^17^
Radiotherapy		Turgut *et al*,^17^ Lo Galbo *et al*,^21^ Qian *et al*^20^	Sinard *et al*,^3^ Léon *et al*,^13^ Lo Galbo *et al*,^10^ Viljoen *et al*,^16^ Tami *et al*,^23^ Turner *et al*^24^
Chemotherapy		Sinard *et al*,^3^ Ho *et al*,^4^ Lo Galbo *et al*,^5,10,21^ Plaat *et al*,^7^ Turgut *et al*,^17^ Qian *et al*^20^	
Neck Dissection		Ho *et al*,^4^ Qian *et al*,^20^ Lo Galbo *et al*,^21^ Plaat *et al*,^7^ Sinard *et al*,^3^ Turgut *et al*^17^	Lo Galbo *et al*^5,10^

#### Demographic factors

**Figure 2:**
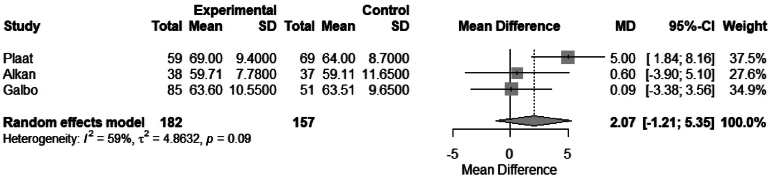
Meta-analysis comparing mean difference in age between euthyroid and hypothyroid patients

**Figure 3:**
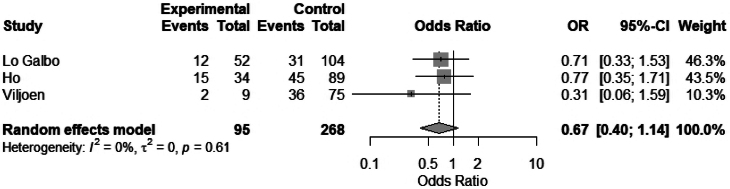
Meta-analysis of age over 70 vs age under 70

The association of hypothyroidism and age was assessed in 11 studies. Of these, three studies had complete data to perform a meta-analysis to evaluate the mean difference in age between euthyroid and hypothyroid patients. No association was found between age and hypothyroidism: mean difference 2.0 (95% CI −1.21 to 5.35), *I*^2^ = 59.3% (Figure 2). Three studies stratified patients by age >70 years, and no significant difference was found in the meta-analysis: odds ratio (OR) 0.67 (95% CI 0.40 to 1.14), *I*^2^ = 0.0% (Figure 3).

**Figure 4:**
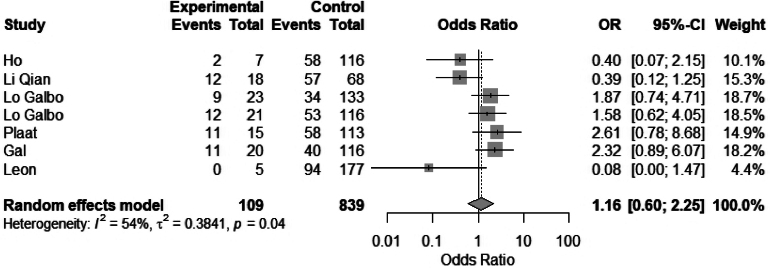
Meta-analysis of female vs male

Sex as a risk factor for hypothyroidism following laryngectomy was evaluated by 11 studies. Seven studies were included in a meta-analysis for sex. No statistically significant difference was found between females and males: OR 1.1 (95% CI 0.6 to 2.25), *I*^2^ = 52 (Figure 4). Gal *et al* found that being female was associated with hypothyroidism and suggested that the relationship between female sex and hypothyroidism could be due to the effect of oestrogen supplements and the risk of subclinical Hashimoto’s thyroiditis in females.^6^ Léon *et al* found a significant difference between females and the development of hypothyroidism in univariate analysis only, possibly because of the increased incidence of thyroid diseases in females.^13^

#### Thyroidectomy

**Figure 5:**
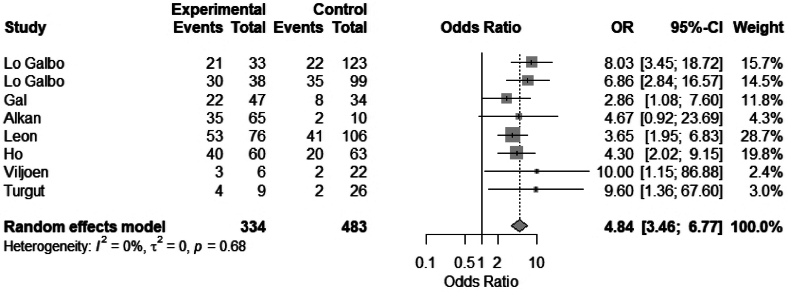
Meta-analysis of hemithyroidectomy vs no thyroidectomy

Eleven studies evaluated the association between hemithyroidectomy and hypothyroidism. A total of eight studies were included in a meta-analysis. Hemithyroidectomy was significantly associated with hypothyroidism following laryngectomy: OR 4.84 (95% CI 3.46 to 6.77), *I*^2^ = 0% (Figure 5). Of the 11 studies, 9 showed a significantly increased risk of hypothyroidism in patients who had concomitant hemithyroidectomy.^4,5,7,10,13–17^

#### Tumour stage

**Figure 6:**
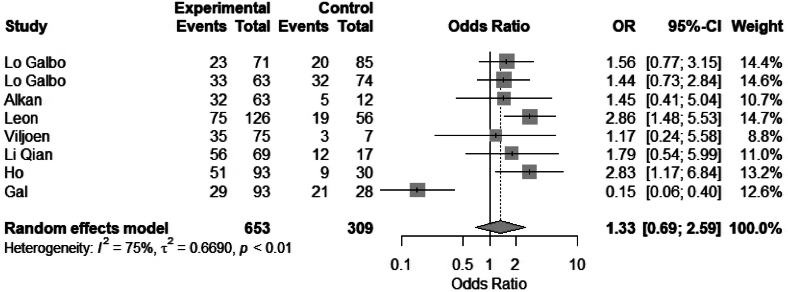
Meta-analysis of early stage vs advanced stage

Eight studies were included in a meta-analysis to compare the association between early (T1, T2) vs advance tumour stage (T3, T4) and hypothyroidism. No significant difference was found in the meta-analysis: OR 1.33 (95% CI 0.69 to 2.59), *I*^2^ = 75% (Figure 6). Three multivariate analyses reported significantly increased rates of hypothyroidism in more advanced tumours.^4,18,19^ Rosko *et al* reported a relative risk of 3.2 for patients with advanced-stage disease (stage 3 or 4) compared with early-stage disease (stage 1 or 2) (95% CI 1.4 to 7.3, *p* = 0.005).^19^ However, nine studies reported no significant difference for stage as a risk factor.^5,10,13,14,16,20–23^ In a multivariate study, Gal *et al* reported that thyroid gland invasion by the tumour increased risk of hypothyroidism.^6^

#### Radiotherapy

**Figure 7:**
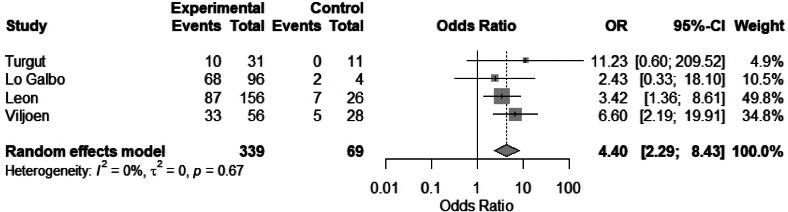
Meta-analysis of radiotherapy vs no radiotherapy

Nine studies evaluated the association between radiotherapy and hypothyroidism; four studies were eligible to be included in the meta-analysis. We found that radiotherapy was significantly associated with hypothyroidism: OR 4.4 (95% CI 2.29 to 8.43), *I*^2^ = 0% (Figure 7). Radiotherapy was identified as having significant associations with hypothyroidism in six studies.^3,10,13,16,23,24^ Three other studies showed no significant difference in radiotherapy treatment.^17,20,21^ Gal *et al* reported that preoperative radiotherapy was associated with a 2.76-fold relative risk (*p* < 0.01) of developing hypothyroidism compared with no radiotherapy, whereas postoperative radiotherapy was associated with a 0.92-fold relative risk (*p* = 0.72). Plaat *et al* found that postoperative radiotherapy alone was associated with a lower risk of developing hypothyroidism (hazard ratio 0.49 [95% CI: 0.28–0.86]) when compared with salvage total laryngectomy after radiotherapy.^7^ Two studies reported a significant dose-dependent relationship between radiotherapy and hypothyroidism.^5,14^

#### Chemotherapy

**Figure 8:**
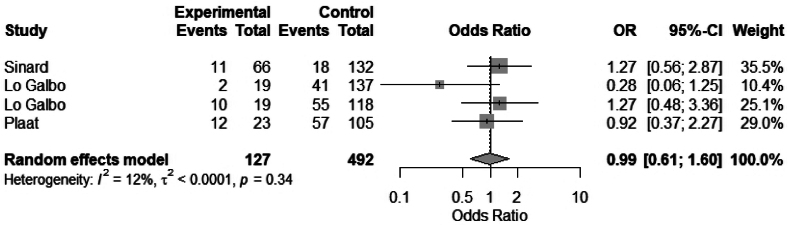
Meta-analysis of chemotherapy vs no chemotherapy

Four studies were included in a meta-analysis to evaluate the association between chemotherapy and hypothyroidism. We found no statistically significant difference: OR 0.9 (95% CI 0.61 to 1.60), *I*^2^ = 12% (Figure 8). No studies found a statistically significant association with chemotherapy in their individual analysis.^3,5,7,10,13,19–21^

#### Neck dissection

**Figure 9:**
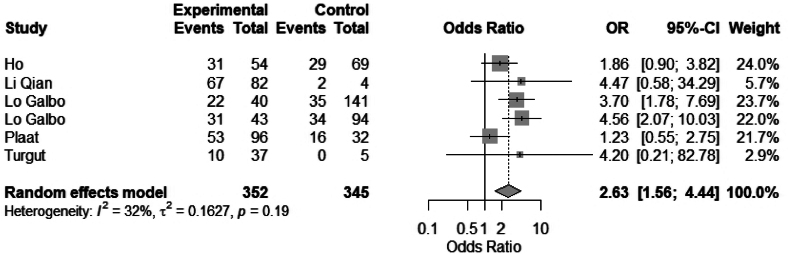
Meta-analysis of neck dissection vs no neck dissection

Eight studies evaluated the association between neck dissection and hypothyroidism.^3–5,7,10,17,20,21^ Seven of these studies were evaluated in a meta-analysis. Neck dissection was significantly associated with hypothyroidism: OR 2.63 (95% CI 1.56 to 4.44), *I*^2^ = 32 (Figure 9). Multivariate analysis by Lo Galbo *et al* showed a statistical significance (*p* = 0.003) that was applicable to both ipsilateral and bilateral neck dissections.^10^ Four studies looked specifically at pretracheal or level VI dissection and risk of hypothyroidism.^10,17,19,21^ Lo Galbo *et al* found that paratracheal lymph node dissection was a predictive factor for developing hypothyroidism (*p* < 0.017).^10^ Univariate regression analysis by Turgut *et al* showed that the risk for hypothyroidism was 31 times higher with level VI dissection (*p* < 0.004) (no confidence interval was stated).

### Prevention

Three studies referred to establishing prevention measures in their stated aims. These studies focused primarily on a demonstration of reduced hypothyroidism without impaired oncologic control when thyroidectomy is targeted to select patient groups rather than applied empirically.^16,25,26^

Primary measures of restricted thyroid dissection in selected cases were advocated in nine studies; preserving the thyroid gland where possible to reduce the risk of hypothyroidism.^15,16,26–32^ El-Sebai Ali *et al* reported no difference in local recurrence rates between patients who had preservation of the thyroid gland and those who did not.^15^ Hemithyroidectomy is recommended in selected cases of subglottic tumours, extra-laryngeal tumours, direct invasion of the thyroid gland, paratracheal nodal metastases and invasion of the paraglottic space.^15,16,26–32^ Attention to preservation of the blood supply to the thyroid gland is recommended as a preventative measure during dissection of contralateral lobe.^1,3,25^

The main secondary prevention recommendation was advocating preoperative (*n* = 4)^6,10,20,24^ and/or postoperative (*n* = 25) thyroid function test (TFT) monitoring. Several studies justified this further, with recommendations of monitoring postoperative TFTs in all treatment modalities.^2,28^ Others recommended monitoring only for patients who underwent combined surgery and radiotherapy or for patients receiving postoperative radiotherapy.^18,33,34,35^ Life-long surveillance of thyroid function was recommended in patients who had any treatment for hypopharyngeal or laryngeal cancer.^17^ The frequency and duration of the suggested surveillance varied.

Ten studies advised treatment of all forms of hypothyroidism.^10,13,17,18,24,25,33–36^ Some suggested empirical prescription of levothyroxine in patients with high risk factors, following laryngectomy, pharyngo-laryngo-oesophagectomy with chemoradiotherapy, or total laryngectomy with thyroid lobectomy and radiotherapy.^3,6,20,37^ Saito *et al* suggested empirical treatment for patients who underwent postoperative radiotherapy to prevent symptomatic endocrine complications.^37^ These studies did not specify the duration of routine thyroxine treatment or assess the long-term outcomes of patients on this treatment.

## Discussion

This systematic review and meta-analysis aim to evaluate the incidence of hypothyroidism following laryngectomy, and identify risk factors and preventative measures. Our study of 40 articles showed an overall incidence of hypothyroidism of 50% (38.3–75.7). From our meta-analysis, the following risk factors were significantly associated with hypothyroidism following laryngectomy: hemithyroidectomy, OR 4.84 (95% CI 3.46 to 6.77); radiotherapy, OR 4.4 (95% CI 2.29 to 8.43); and neck dissection, OR 2.63 (95% CI 1.56 to 4.44). The preventative strategies of hypothyroidism observed in our study included restricted thyroid dissection in selected cases, preservation of blood supply to the thyroid gland during dissection, preoperative TFTs and postoperative TFT monitoring, including in those with subclinical hypothyroidism.^1,3,15,16,25–32,^

The updated search retrieved the study by Nasser *et al*, which reported hypothyroidism in 45% of patients following total laryngectomy,^38^ comparable with our findings. The Nasser *et al* study also showed a significant association between adjuvant radiotherapy (*p* = 0.001) and central neck dissection (*p* = 0.007) with the development of postoperative hypothyroidism.

This systematic review confirmed variability in the reported incidence of hypothyroidism after laryngectomy, with heterogeneity noted in patient characteristics and treatment modalities. Definitions of hypothyroidism varied between the 40 studies. Half of the reports used both TSH and fT4 levels. Nine studies used only TSH levels.^2,3,7,15,18,19,27,37,39^ Eleven studies reported rates of hypothyroidism without defining levels that meet the diagnosis of hypothyroidism.^1,6,26,28–32,40–42^ The wide incidence range for hypothyroidism of 38.3–75.7% is likely attributed to variability in the definition used and surgical extent. Timings of ascertainment of hypothyroidism and the length of follow-up were also variable or not reported in the studies.

The British Thyroid Association defined overt primary hypothyroidism as TSH ≥ 10mU/L with fT4 levels below the reference range, whereas subclinical hypothyroidism was defined as TSH of 5–10mU/, but fT4 levels in the reference range.^43^ Standardising the definition of clinical hypothyroidism and subclinical hypothyroidism is important to allow appropriate comparisons and summation of outcomes.

We would recommend increasing the number of high-quality studies aiming to formally assess the efficacy of prevention measures, such as the timing and targeting of postoperative testing or the effects of thyroid replacement on patient outcomes.

From our review, it is evident that the treatment of subclinical hypothyroidism is variable between these studies. A systematic review reported no improvement in survival or cardiovascular morbidity following levothyroxine replacement therapy for asymptomatic subclinical hypothyroidism.^44^ Although this was acknowledged by Lo Galbo *et al*, the study recommended screening for hypothyroidism with subsequent treatment if indicated owing to the low burden of this therapy.^10^

We recommend that patients should be monitored for hypothyroidism annually during long-term follow-up to prevent the progression to overt hypothyroidism. In patients treated with external radiotherapy, TFT should be performed every 12 months, because subclinical hypothyroidism can be present for many years before developing into overt hypothyroidism.^45^

Initial timing of the ascertainment of hypothyroidism is important because measurement in the early postoperative period may not reflect true hypothyroidism. The gold standard measure is free T4 measurements to ascertain hypothyroidism because TSH is influenced by a range of medical factors. As recommended by the British Thyroid Association report, where a diagnosis of overt hypothyroidism cannot be confirmed, clinicians should trial without levothyroxine with a repeat serum TSH after 6 weeks. Emphasis should be made to check TFTs following discharge from hospital to prevent undiagnosed overt or subclinical hypothyroidism.^43^

Management of primary hypothyroidism by the British Thyroid Association states that synthetic levothyroxine is the treatment of choice in hypothyroidism. TSH levels should be monitored 6–8 weekly with dose adjusted accordingly. Following stabilisation of TSH levels, this can be checked 4–6 monthly, and then annually.^46^

The evidence base underpinning the timing and efficacy of postoperative testing of hypothyroidism and any subsequent treatment is lacking despite this being common practice in most reporting institutions. A standardised protocol for the identification, prevention and treatment, and follow-up of hypothyroidism specifically in laryngectomy patients is recommended.

### Study limitations

Limitations of this systematic review include the heterogeneity of the study population with regards to disease extent and treatment modalities. Furthermore, some studies did not exclude preoperative hypothyroidism, and thus reported rates of hypothyroidism post laryngectomy may be overestimated. From our quality assessment, 39 of the 40 studies were observational with low to moderate quality. There was only one randomised controlled trial.^15^ In addition, we excluded articles not available in the English language because formal translation and analysis was beyond the scope of this review.

## Conclusions

Our systematic review of 40 studies showed that a significant proportion of patients develop hypothyroidism following laryngectomy. Commonly recognised risk factors of developing hypothyroidism are hemithyroidectomy, radiotherapy and neck dissection. This review shows the lack of data on preventative measures. Utilising these known risk factors may direct a consensus to prevent and manage hypothyroidism. Further well-designed multicentre observational studies are needed to design a protocol for managing hypothyroidism following laryngectomy.

## Author contributions

Conceptualisation and methodology, EW and OE; literature search, EW; data entry and analyses, JYT and EW; original drafting of manuscript, JYT; review and revision of manuscript, JYT and OE. All authors have read and agreed to the published version of the manuscript.

## References

[1] Palmer BV, Gaggar N, Shaw HJ. Thyroid function after radiotherapy and laryngectomy for carcinoma of the larynx. *Head Neck Surg* 1981; **4**: 13–15. 7287444 10.1002/hed.2890040105

[2] Buisset E, Leclerc L, Lefebvre JL *et al.* Hypothyroidism following combined treatment for hypopharyngeal and laryngeal carcinoma. *Am J Surg* 1991; **162**: 345–347. 1951886 10.1016/0002-9610(91)90145-4

[3] Sinard RJ, Tobin EJ, Mazzaferri EL *et al.* Hypothyroidism after treatment for nonthyroid head and neck cancer. *Arch Otolaryngol Head Neck Surg* 2000; **126**: 652–657. 10807335 10.1001/archotol.126.5.652

[4] Ho AC, Ho WK, Lam PK *et al.* Thyroid dysfunction in laryngectomees–10 years after treatment. *Head Neck* 2008; **30**: 336–340. 17636544 10.1002/hed.20693

[5] Lo Galbo AM, de Bree R, Kuik DJ *et al.* The prevalence of hypothyroidism after treatment for laryngeal and hypopharyngeal carcinomas: are autoantibodies of influence? *Acta Otolaryngol* 2007; **127**: 312–317. 17364370 10.1080/00016480600818096

[6] Gal RL, Gal TJ, Klotch DW, Cantor AB. Risk factors associated with hypothyroidism after laryngectomy. *Otolaryngol Head Neck Surg* 2000; **123**: 211–217. 10964293 10.1067/mhn.2000.107528

[7] Plaat RE, van Dijk BAC, Muller Kobold AC *et al.* Onset of hypothyroidism after total laryngectomy: effects of thyroid gland surgery and preoperative and postoperative radiotherapy. *Head Neck* 2020; **42**: 636–644. 31833166 10.1002/hed.26048 PMC7154538

[8] Chaker L, Bianco AC, Jonklaas J, Peeters RP. Hypothyroidism. *Lancet* 2017; **390**: 1550–1562. 28336049 10.1016/S0140-6736(17)30703-1 PMC6619426

[9] Shafer RB, Nuttall FQ, Pollak K, Kuisk H. Thyroid function after radiation and surgery for head and neck cancer. *Arch Intern Med* 1975; **135**: 843–846. 1130929

[10] Lo Galbo AM, Kuik DJ, Lips P *et al.* A prospective longitudinal study on endocrine dysfunction following treatment of laryngeal or hypopharyngeal carcinoma. *Oral Oncol* 2013; **49**: 950–955. 23602256 10.1016/j.oraloncology.2013.03.450

[11] Murad MH, Sultan S, Haffar S, Bazerbachi F. Methodological quality and synthesis of case series and case reports. *BMJ Evid Based Med* 2018; **23**: 60–63. 10.1136/bmjebm-2017-110853 PMC6234235 29420178

[12] Higgins JPT, Altman DG, Gøtzsche PC *et al.* The Cochrane collaboration’s tool for assessing risk of bias in randomised trials. *BMJ* 2011; **343**: d5928. 22008217 10.1136/bmj.d5928 PMC3196245

[13] Léon X, Gras JR, Pérez A *et al.* Hypothyroidism in patients treated with total laryngectomy. A multivariate study. *Eur Arch Otorhinolaryngol* 2002; **259**: 193–196. 12064507 10.1007/s00405-001-0418-x

[14] Alkan S, Baylancicek S, Ciftçic M *et al.* Thyroid dysfunction after combined therapy for laryngeal cancer: a prospective study. *Otolaryngol Head Neck Surg* 2008; **139**: 787–791. 19041504 10.1016/j.otohns.2008.09.011

[15] El-Sabai Ali M, Ebada HA, El-Shaheed MA *et al.* Routine thyroidectomy with total laryngectomy: is it really indicated? A randomized controlled trial. *Ann Med Surg (Lond)* 2022; **74**: 103309. 35145675 10.1016/j.amsu.2022.103309 PMC8818527

[16] Viljoen G, McGuire JK, Alhadad A *et al.* Does thyroid-sparing total laryngectomy decrease the risk of hypothyroidism? *J Laryngol Otol* 2020; **134**: 1069–1072. 33243316 10.1017/S0022215120002479

[17] Turgut OK, Erişen L, Coşkun H *et al.* Hypothyroidism after primary surgical treatment for laryngeal and hypopharyngeal cancer. *Kulak Burun Bogaz Ihtis Derg* 2008; **18**: 125–130. 18984992

[18] Garcia-Serra A, Amdur RJ, Morris CG *et al.* Thyroid function should be monitored following radiotherapy to the low neck. *Am J Clin Oncol* 2005; **28**: 255–258. 15923797 10.1097/01.coc.0000145985.64640.ac

[19] Rosko AJ, Birkeland AC, Bellile E *et al.* Hypothyroidism and wound healing after salvage laryngectomy. *Ann Surg Oncol* 2018; **25**: 1288–1295. 29264671 10.1245/s10434-017-6278-4 PMC6002868

[20] Qian LL, Hopkins ME, Nixon IJ, Hay A. Thyroid function post laryngectomy and hemithyroidectomy – do all laryngectomy patients need thyroid replacement? *Clin Otolaryngol* 2022; **47**: 323–327. 34698445 10.1111/coa.13883

[21] Lo Galbo AM, de Bree R, Kuik DJ *et al.* Paratracheal lymph node dissection does not negatively affect thyroid dysfunction in patients undergoing laryngectomy. *Eur Arch Otorhinolaryngol* 2010; **267**: 807–810. 19915857 10.1007/s00405-009-1152-z

[22] Ozawa H, Saitou H, Mizutari K *et al.* Hypothyroidism after radiotherapy for patients with head and neck cancer. *Am J Otolaryngol* 2007; **28**: 46–49. 17162132 10.1016/j.amjoto.2006.06.011

[23] Tami TA, Gomez P, Parker GS *et al.* Thyroid dysfunction after radiation therapy in head and neck cancer patients. *Am J Otolaryngol* 1992; **13**: 357–362. 1443391 10.1016/0196-0709(92)90076-6

[24] Turner SL, Tiver KW, Boyages SC. Thyroid dysfunction following radiotherapy for head and neck cancer. *Int J Radiat Oncol Biol Phys* 1995; **31**: 279–283. 7836081 10.1016/0360-3016(93)E0112-J

[25] Kojima R, Tsukahara K, Motohashi R *et al.* Extent of thyroid resection and thyroid function after postoperative radiotherapy following total laryngectomy or total pharyngo-laryngo-esophagectomy. *Int J Clin Oncol* 2017; **22**: 438–441. 28054142 10.1007/s10147-016-1082-x

[26] Yuen AP, Wei WI, Lam KH, Ho CM. Thyroidectomy during laryngectomy for advanced laryngeal carcinoma–whole organ section study with long-term functional evaluation. *Clin Otolaryngol Allied Sci* 1995; **20**: 145–149. 7634521 10.1111/j.1365-2273.1995.tb00032.x

[27] Al-Khatib T, Mendelson AA, Kost K *et al.* Routine thyroidectomy in total laryngectomy: is it really indicated? *J Otolaryngol Head Neck Surg* 2009; **38**: 564–567. 19769827

[28] Biel MA, Maisel RH. Indications for performing hemithyroidectomy for tumors requiring total laryngectomy. *Am J Surg* 1985; **150**: 435–439. 4051106 10.1016/0002-9610(85)90149-7

[29] Gürbüz MK, Açikalin M, Tasar S *et al.* Clinical effectiveness of thyroidectomy on the management of locally advanced laryngeal cancer. *Auris Nasus Larynx* 2014; **41**: 69–75. 24176487 10.1016/j.anl.2013.10.004

[30] Mourad M, Saman M, Sawhney R, Ducic Y. Management of the thyroid gland during total laryngectomy in patients with laryngeal squamous cell carcinoma. *Laryngoscope* 2015; **125**: 1835–1838. 26059344 10.1002/lary.25263

[31] Nasehi A, Abbaszadeh HA, Ahmady Roozbahany N *et al.* Thyroid gland involvement and the efficiency of thyroidectomy in patients having larynx and hypopharyngeal cancers treated with surgery. *Int J Cancer Manag* 2019; **12**: e88750.

[32] Panda S, Kumar R, Konkimalla A *et al.* Rationale behind thyroidectomy in total laryngectomy: analysis of endocrine insufficiency and oncological outcomes. *Indian J Surg Oncol* 2019; **10**: 608–613. 31857751 10.1007/s13193-019-00935-4 PMC6895295

[33] Donnelly MJ, O’Meara N, O’Dwyer TP. Thyroid dysfunction following combined therapy for laryngeal carcinoma. *Clin Otolaryngol Allied Sci* 1995; **20**: 254–257. 7554340 10.1111/j.1365-2273.1995.tb01861.x

[34] Posner MR, Ervin TJ, Miller D *et al.* Incidence of hypothyroidism following multimodality treatment for advanced squamous cell cancer of the head and neck. *Laryngoscope* 1984; **94**: 451–454. 6708688 10.1288/00005537-198404000-00002

[35] Smolarz K, Malke G, Voth E *et al.* Hypothyroidism after therapy for larynx and pharynx carcinoma. *Thyroid* 2000; **10**: 425–429. 10884190 10.1089/thy.2000.10.425

[36] Negm H, Mosleh M, Fathy H, Awad A. Thyroid and parathyroid dysfunction after total laryngectomy in patients with laryngeal carcinoma. *Eur Arch Otorhinolaryngol* 2016; **273**: 3237–3241. 27225281 10.1007/s00405-016-4105-3

[37] Saito Y, Kawakubo H, Takami H *et al.* Thyroid and parathyroid functions after pharyngo-laryngo-esophagectomy for cervical esophageal cancer. *Ann Surg Oncol* 2019; **26**: 3711–3717. 31187362 10.1245/s10434-019-07476-8

[38] Nassar AA, Shoaib AA, Dewidar HM, Azooz KO. Incidence of post total laryngectomy hypothyroidism: effects of thyroid gland surgery and post-operative radiotherapy. *Indian J Otolaryngol Head Neck Surg* 2023; **75**: 1336–1343. 37636632 10.1007/s12070-023-03562-2 PMC10447349

[39] Widström A, Carstam L, Odelberg-johnson O, Widström A. Parathyroid and thyroid function after treatment of cancer of the larynx. *Acta Otolaryngol* 1982; **93**: 212–214.

[40] Amusa YB, Badmus TA, Olabanji JK, Oyebamiji EO. Laryngeal carcinoma: our experience at Obafemi Awolowo University teaching hospital complex, Ile-Ife, Nigeria. *Cent Afr J Med* 2009; **55**: 54–58. 21977845 10.4314/cajm.v55i9-12.63641

[41] Jayasuriya C, Dayasiri MB, Indranath N *et al.* The quality of life of laryngectomised patients. *Australasian Medical Journal* 2010; **3**: 353–357.

[42] Larbcharoensub N, Wattanatranon D, Leopairut J *et al.* Clinicopathologic findings and treatment outcome of laryngectomized patients with laryngeal cancer and hypopharyngeal cancer: an experience in Thailand. *Asian Pac J Cancer Prev* 2017; **18**: 2035–2042. 28843218 10.22034/APJCP.2017.18.8.2035 PMC5697456

[43] Ahluwalia R, Baldeweg SE, Boelaert K *et al.* Use of liothyronine (T3) in hypothyroidism: joint British thyroid association/society for endocrinology consensus statement. *Clin Endocrinol (Oxf)* 2023; **99**: 206–216. 37272400 10.1111/cen.14935

[44] Villar HC, Saconato H, Valente O, Atallah AN. Thyroid hormone replacement for subclinical hypothyroidism. *Cochrane Database Syst Rev* 2007; **2007**: CD003419. 17636722 10.1002/14651858.CD003419.pub2 PMC6610974

[45] British Thyroid Association. *UK Guidelines for the use of thyroid function tests*. https://www.british-thyroid-association.org/sandbox/bta2016/uk_guidelines_for_the_use_of_thyroid_function_tests.pdf (cited May 2024).

[46] Okosieme O, Gilbert J, Abraham P *et al.* Management of primary hypothyroidism: statement by the British Thyroid Association Executive Committee. *Clin Endocrinol (Oxf)* 2016; **84**: 799–808. 26010808 10.1111/cen.12824

